# Versatile Polypropylene Composite Containing Post-Printing Waste

**DOI:** 10.3390/polym14245335

**Published:** 2022-12-07

**Authors:** Krzysztof Moraczewski, Tomasz Karasiewicz, Alicja Suwała, Bartosz Bolewski, Krzysztof Szabliński, Magdalena Zaborowska

**Affiliations:** 1 Faculty of Materials Engineering, Kazimierz Wielki University, Chodkiewicza 30 Str., 85-064 Bydgoszcz, Poland; 2 Blue System Sp. z o.o., Rynkowska 17D Str., 85-503 Bydgoszcz, Poland

**Keywords:** circular economy, polypropylene, post-printing waste

## Abstract

The paper presents the results of the research on the possibility of using waste after the printing process as a filler for polymeric materials. Remains of the label backing were used, consisting mainly of cellulose with glue and polymer label residue. The properly prepared filler (washed, dried, pressed and cut) was added to the polypropylene in a volume ratio of 2:1; 1:1; 1:2; and 1:3 which corresponded to approximately 10, 5, 2.5 and 2 wt % filler. The selected processing properties (mass flow rate), mechanical properties (tensile strength, impact strength, dynamic mechanical analysis) and thermal properties (thermogravimetric analysis, differential scanning calorimetry) were determined. The use of even the largest amount of filler did not cause disqualifying changes in the determined properties. The characteristics of the obtained materials allow them to be used in various applications while reducing costs due to the high content of cheap filler.

## 1. Introduction

Wastes are substances or objects resulting from human activity, as well as residues from their production, which are intended for disposal. The proper management of waste and by-products from industrial and natural production and activities (agriculture, horticulture and others) is the key to sustainable development, reduction in pollution, increase in storage space, minimization of landfill, reduction in energy consumption and facilitation of the circular economy [1].

The circular economy is a regenerative economic system in which the consumption of raw materials and the amount of waste as well as the emission and loss of energy are minimized by creating a closed loop of processes in which waste from one process is used as raw materials for others, which minimizes the amount of waste production [2,3]. Waste management is a series of processes related to the collection, transport and processing, including the supervision of this type of activity, as well as the subsequent handling of waste disposal sites, as well as activities related to waste trading [4,5].

One of the possibilities of reusing post-production waste is its use for the production of other materials, including the production of composite materials with various matrices. In recent years, composites with a polymer matrix and post-production waste fillers or reinforcements have attracted a lot of interest. Due to the characteristics of polymeric materials, thermoplastics in particular, it is possible to easily add post-production wastes in various forms to the polymer mass and subsequently easily process them through extrusion and injection processes.

In the production of polymer composites, the post-production waste used can be divided into two types. The first consists of organic wastes from the agricultural or food industry. The second consists of inorganic wastes from heavy industry such as the steel industry or petrochemical industry [1].

The most frequently used post-production waste materials in the production of polymer composites are natural wastes. Natural wastes are cheap and easily renewable, and their biodegradability is one of their most important features. Among the organic waste, the most commonly used post-production wastes is wood obtained from the wood and furniture industries. Post-production wastes in the form of flour or chips from the processes of cutting, turning, milling, planing, etc. are used as fillers for the production of wood-polymer composites (WPC), most often with polyolefin or poly(vinyl chloride) matrix, but also biodegradable polymers such as polylactide [6,7,8,9,10,11]. 

Other organic post-production wastes used in the production of polymer composites include: shells of nuts and seeds [12,13,14,15], bamboo [16,17,18,19], coconut shells [20,21,22,23,24], husk of rice and cereals [25,26,27,28,29,30], fruit and vegetables pomace [31,32,33,34], as well as waste of animal origin, such as egg shells or shells of crustaceans [35,36,37,38].

In most cases, these organic fillers can be easily integrated into thermoplastic or thermoset matrices to change their thermal, mechanical and tribological properties. However, not always only favorable changes in properties are observed. It is very often necessary to properly modify the waste in order to obtain materials with good performance parameters. Therefore, the deterioration of properties, especially mechanical properties, can most often be observed after adding unmodified waste. However, this deterioration does not have to be disqualifying for these composites, as very often, their properties are still sufficient for many planned applications, and their great advantage is a much lower price and environmental friendliness thanks to the use of cheap waste materials.

Printing houses, publishing houses and printing companies have to tackle the issue of increasing amounts of post-production waste on a daily basis. In the printing industry, the different types of waste vary significantly. The most important of the solid, liquid and gaseous wastes produced in the printing industry before, during and after the printing process are waste ink, ink sludge and solvents emerging after machine washing, wastewater of water-based ink, plate and film developer and fixer solutions, cleaning solvents and volatile organic compounds (VOCs) [39]. The printing industry generates large amounts of scrap paper, catalogs, posters, cardboard boxes and plastic foil. The loose waste piles up and is stored in warehouses, taking up space and making it difficult to move around [40]. Some of the produced wastes even fall into the hazardous waste category due to their processing characteristics during the production process. The effective and regular extermination of these wastes is necessary to protect the environment. This can be provided only by the application of waste management. Some wastes are recycled and reused at printing industry, but in some cases, that recycling is impossible, and these wastes should be eliminated without harming human health and the environment [41]. In particular, the materials whose disposal is compulsory should be classified at the source and sent to licensed disposal companies. One of the solutions may be the reuse of generated waste in the production of other materials while being part of the circular economy.

The paper presents the results of research on the possibility of using unmodified post-production waste from the food label printing process. In the product labeling process, the liner is often overlooked by many brands as part of the waste stream. Despite the various recycling programs available on the market, such as UPM Raflatac’s RafCycle^®^, they can still be a major environmental problem. The process of cutting the underlay to the production dimensions still produces from several dozen to several hundred kilograms of cut waste, which does not qualify for the recycling program and requires proper disposal, which is a problem for the company. Due to the lack of modification of the waste, it was expected that the properties of the obtained composites would deteriorate in relation to pure polymer. However, it is purposeful to check whether the properties of the new composites will still be sufficient for the use of the obtained composites.

## 2. Materials and Methods

The matrix of the tested materials was polypropylene (PP) Moplen EP548U (LyondellBasell, Rotterdam, The Netherlands). The basic properties of the polymer according to the data sheet are:

- Melt Flow Rate (230 °C/2.16 kg): 70 g/10 min.
- Density: 0.90 g/cm^3^.
- Tensile Stress at Yield: 28 MPa.
- Tensile Strain at Break: 30%.
- Tensile Strain at Yield: 5%.
- Charpy Impact Strength (Notched): 4 kJ/m^2^.
- Tensile Modulus: 1450 MPa.
- Melt temperature: 160 °C.

The filler of new materials is post- production waste after the printing process in the form of cut off yellow transparent glassine backing cellulose paper with the trade name HONEY GLASSINE 65 (UPM Raflatac, Tempere, Finland) with possible residues of hotmelt rubber permanent adhesive and a polymer (polypropylene or polyethylene) label. Glassine is a smooth and glossy paper that is air, water, and grease resistant. It is usually available in densities between 50 and 90 g/m^2^. It is translucent unless dyes are added to color it or make it opaque. Product is designed for general purpose high-quality multicolor-printed labels to packaged food and homecare applications in ambient conditions. It is intended for all reelstock applications, and it is suitable for automatic dispensing. The basic properties of the backing paper according to the data sheet are:

- Substance: 55 g/m^2^.
- Caliper: 49 µm.
- Tensile strength MD: 6.0 kN/m.
- Tensile strength CD: 2.3 kN/m.
- Transparency: 49%.

**Figure 1:**
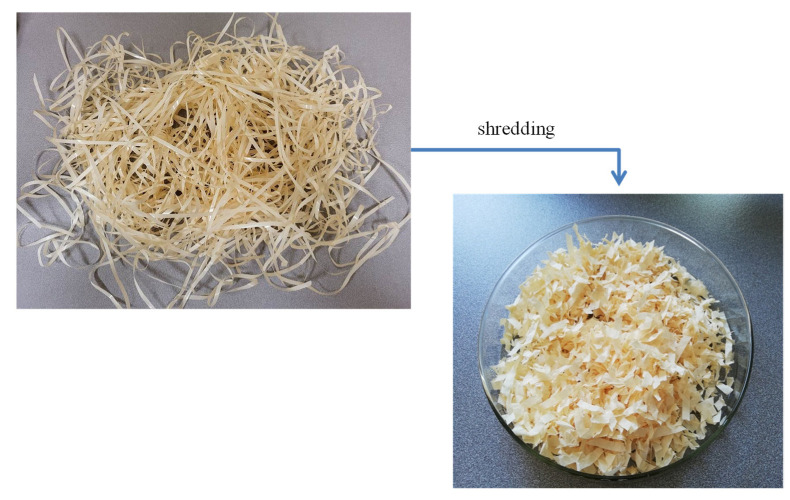
Initial and final form of the filler.

The input material from the production of the filler was in the form of long strands, approx. 4 mm wide. Initially, the waste was soaked in water, and then, it was formed with a hydraulic press into discs with a diameter of about 10 cm. Then, the discs were dried in a laboratory dryer and comminuted using a laboratory grinder. Ultimately, the filler was in the form of short fragments with a width and thickness similar to the original strips (Figure 1).

The prepared filler was added to the polymer matrix in a 1:3; 1:2; 1:1 and 2:1 volume ratio, which corresponded to mass concentrations at the level of 1.9; 2.5; 5.1 and 10.3 wt %. The test samples are marked with the symbols P_x, where x is the volume ratio of the filler. All test results were compared to the results of a pure polypropylene sample, which was abbreviated as PP. Sample designations with corresponding volume ratio of filler and calculated mass concentration are presented in Table 1.

The granules of individual composites were obtained from the prepared composite masterbatches by extrusion. Extrusion was carried out on a W25-30D single screw extruder (Metalchem, Toruń, Poland). In order to obtain a very thorough mixing of both components, intensive mixing screws were used for this purpose, which additionally included kneading and retracting segments. The rotational speed of the screw was constant at 200 rpm. The temperatures of individual zones of the extruder were: 175, 185, 195 and 195 °C, and the temperature of the head was 120 °C. The material coming out of the head was cooled in a bath with water and then cut with a knife granulator.

**Figure 2:**
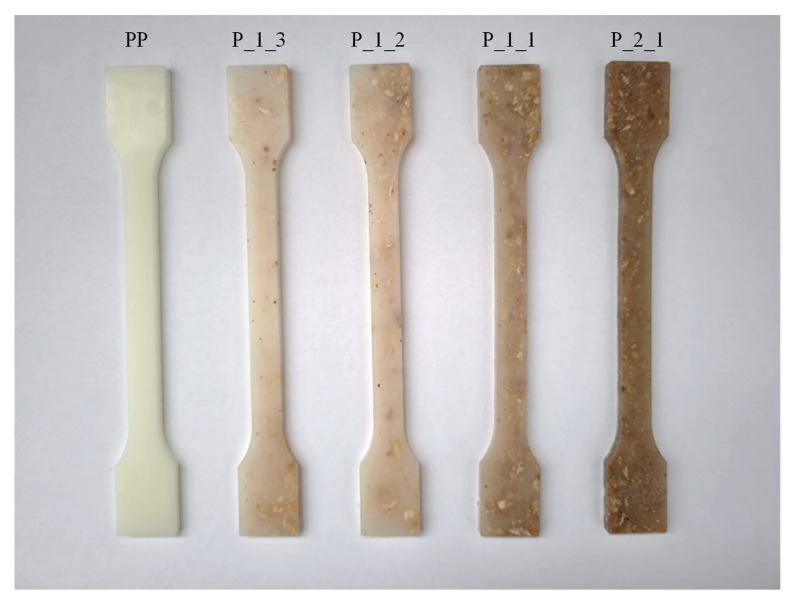
Test specimens obtained by injection method.

From the obtained granulate by injection method, test specimens were obtained in the form of standardized bone-shaped samples (Figure 2) and bars. Injection molding was carried out on a TRX 80 Eco (Tederic, Zhejiang, China) injection molding machine. The injection molding process for all compositions was conducted under the following conditions: 170 °C, 170 °C and 175 °C with head temperature—180 °C. Other parameters follow: mold temperature—35 °C, injection pressure—35 bar, cooling time—30 s.

Melt flow rate (MFR) studies were performed using an MP600 plastometer (Tinius Olsen, Horsham, PA, USA). The tests were carried out at a temperature of 190 °C with a piston load of 2.16 kg. For each tested material, 12 measurement sections were obtained of which 10 values were taken for the calculations (two extreme ones were rejected).

The mechanical properties of the tested materials were determined by the tensile strength test and the un-notch impact test. For the mechanical tests, twelve samples of each composition were used for each test. The result of mechanical tests are the values obtained as the arithmetic mean of individual parameters together with the calculated values of the standard deviation.

Static tensile tests on PP and filler-containing polymer samples were performed on an Instron 3367 (Instron, Norwood, MA, USA) universal testing machine. The tensile speed was 50 mm/min. As part of the test, the tensile strength (σ_M_), stress at break (σ_B_), strain at maximum stress (ε_M_) and strain at break (ε_B_) were determined.

Charpy impact tests were carried out with an XJ 5Z impact hammer (Liangong, Shandong, China) using a 2 J hammer with a fall velocity of 2.9 m/s. Samples in the form of bars with dimensions of 80 mm × 10 mm × 4 mm were tested. As part of the study, the value of the impact strength without notch (_ua_) was determined.

Thermomechanical (DMA) tests were carried out using a Q800 (TA Instruments, New Castle, DE, USA) dynamic mechanical analyzer. The tests were carried out in the temperature range from 30 to 150 °C with a heating rate of 3 °C/min. The samples were bars with dimensions of 80 mm × 10 mm × 4 mm. The strain was 15 μm, and the strain frequency was 1 Hz.

The Q200 (TA Instruments, New Castle, DE, USA) calorimeter was used in the differential scanning calorimetry (DSC) studies. Samples weighing about 4 mg were heated in the temperature range from 0 to 700 °C with the temperature change rate of 10 °C min^−1^. The test was conducted in a nitrogen atmosphere. Based on the cooling and heating curves, the glass transition temperature (T_g_), the cold crystallization temperature (T_cc_), the change of the cold crystallization enthalpy (ΔH_cc_), the melting point (T_m_), the change of the melting enthalpy (ΔH_cc_) and the degree of crystallinity (X_c_) were determined. X_c_ values were calculated from equation:

(1) $$X_{c}=\left(\frac{\Delta H_{m}-\Delta H_{\mathrm{cc}}}{\Delta H_{m100\%}\cdot \;\left(1-x\right)}\right)\cdot 100\%$$

where ΔH_m100%_—enthalpy change of 100% crystalline PP; 207 J/g [42]. x—share of post-printing waste.

Thermogravimetric analysis (TGA) studies were performed under nitrogen atmosphere using a Q500 thermobalance (TA Instruments, New Castle, DE, USA). The samples weighing about 21 mg were tested in the temperature range from 25 to 700 °C with the temperature change rate of 10 °C/min. Based on the thermogravimetric curves, the values of T_5%_, T_50%_ and T_95%_ were determined, corresponding to the loss temperature of 5%, 50% and 95% of the initial mass of the sample. The value of T_5%_ was adopted as the parameter defining the thermal resistance of the material. From the differential thermogravimetric curve (DTG) (the first derivative of the TG curve), the T_max_ values were also determined, defining the temperatures of the fastest mass loss in the individual degradation stages.

## 3. Results

**Figure 3:**
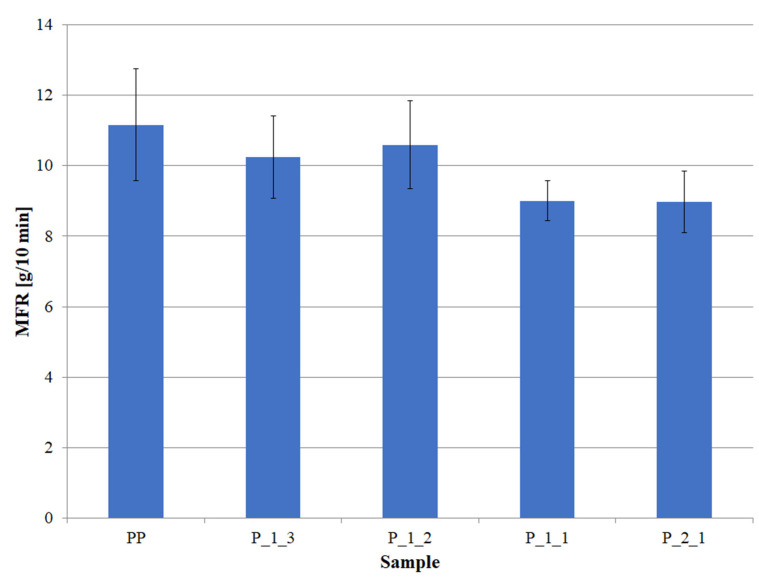
Melt flow rate (MFR) of individual samples.

The determined melt flow rate (MFR) of pure PP was 11.2 g/10 min and was consistent with the literature data (Figure 3). The addition of the filler into the polymer matrix did not cause major changes in the MFR values. Although the applied post-print waste limited the flow of the polymer, which is typical for this type of filler, the obtained decrease in MFR was acceptable. At lower filler contents, i.e., samples P_1_3 and P_1_2, the MFR decreased to the value of about 10 g/10 min. Even a further increase in the amount of filler did not cause a large decrease in MFR, and the recorded values for the samples P_1_1 and P_2_1 were 9.0 g/10 min. Thus, the total decrease in MFR after adding the greatest amount of filler, i.e., twice the volume excess of filler, was 2.2 g/10 min, which is 20% of the value of pure PP.

**Figure 4:**
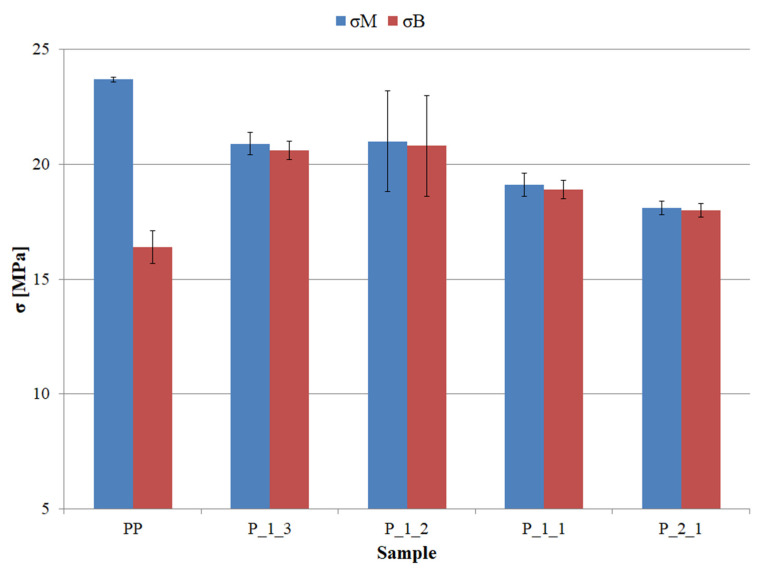
Tensile strength (σ_M_) and stress at break (σ_B_) of individual samples.

The determined tensile strength (σ_M_) and tensile stress (ε_B_) of pure PP samples were slightly lower than the values given in the data sheet for the tested polymer but in line with the literature data for PP. The addition of post-printing waste to the matrix resulted in a slight decrease in the strength of the tested materials (Figure 4) as well as equating the values of σ_M_ and ε_B_, which is related to the change in the elasticity of PP after introducing the filler particles (stress aggregation sites). As for MFR, one observes a clear two-stage decrease in the tensile strength of the tested materials. A smaller drop in strength by approx. 2.5 MPa compared to pure PP was observed for P_1_3 and P_1_2 materials, i.e., materials with a predominance of polymer in the composition. A greater decrease in strength by about 4.5 MPa in relation to pure PP was observed for higher waste content, i.e., P_1_1 and P_2_1 materials, where the initial volume fraction of waste was equal to or higher than the polymer fraction. The overall decrease in the tensile strength of PP after adding the post-printing waste was 4.5 MPa, which is approx. 19% of the value of pure polymer.

**Figure 5:**
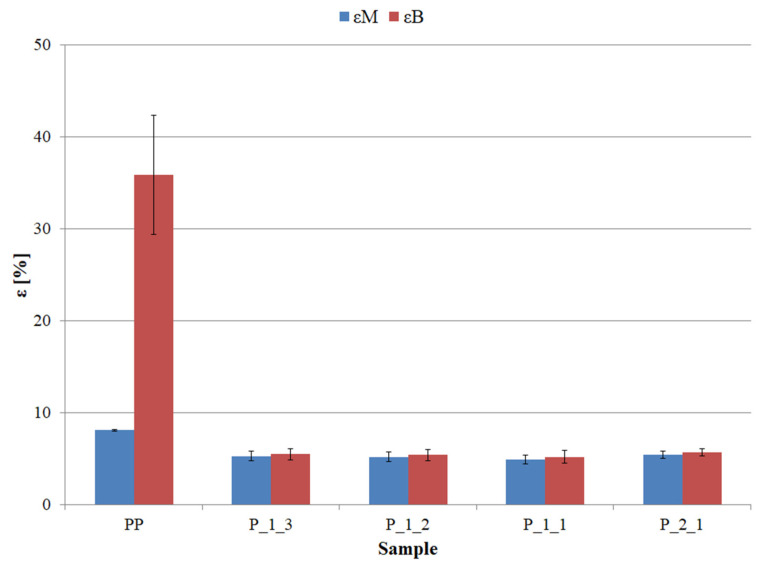
Elongation at maximum stress (ε_M_) and elongation at break (ε_B_) of individual samples.

The introduction of the printing waste into the PP matrix was also accompanied by a large decrease in elongation at break (ε_B_), which was additionally equal to the values of elongation at maximum stress (ε_M_) (Figure 5). Due to the characteristics of PP and the occurrence of the phenomenon of necking during the tensile test, where the stretching and ordering of macromolecules occurs, this polymer is characterized by high ε_B_ values, which significantly exceed the ε_M_ values. The applied printing waste reduces the values of ε_M_ and ε_B_ to the level of approx. 5%, while the values for pure PP were, respectively, 8.1 and 35.9%. The obtained strain drop was the same regardless of the volumetric content of the filler in the matrix. The lack of differences in the deformation between individual materials is probably because, regardless of the amount of waste in the cross-section of the sample, there will always be a filler particle, on which stress aggregation and sample rupture will occur before the necking phenomenon occurs. 

**Figure 6:**
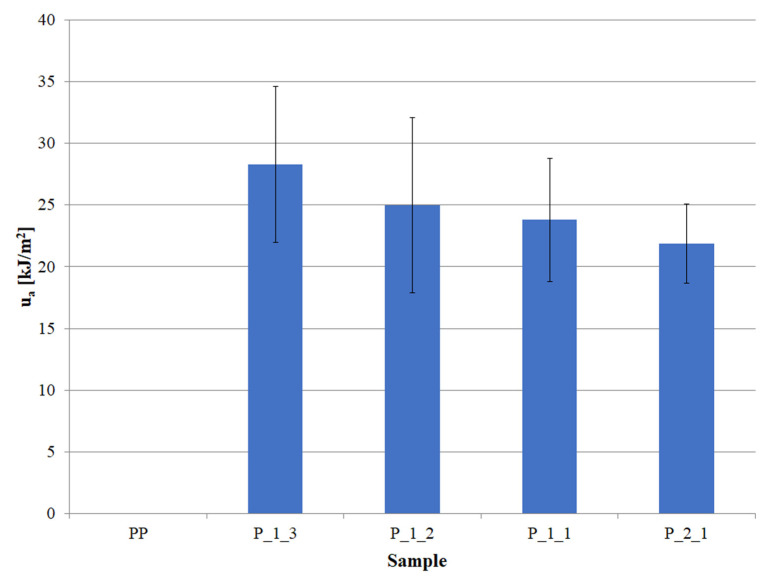
The impact strength (u_a_) of individual samples.

The PP sample subjected to impact tests does not break, which is often observed for pure polypropylene due to its high flexibility and hence high impact resistance. Even the lowest content of post-printing waste in the material caused the samples to break as a result of the impact, and the value of the impact strength was recorded (u_a_) (Figure 6).

**Figure 7:**
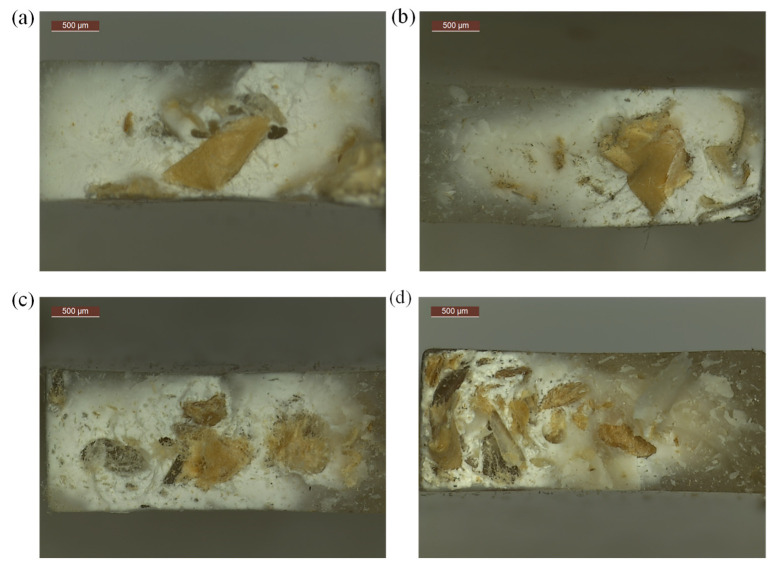
Microscopic photos of the cross-sections of (**a**) P_1_3, (**b**) P_1_2, (**c**) P_1_1, (**d**) P_2_1 sample.

The obtained u_a_ value for the P_1_3 sample was 28.3 kJ/m^2^. With an increase in the filler content, the impact strength decreased, reaching the lowest value of 21.9 kJ/m^2^ for the P_2_1 sample, i.e., a material with a waste content twice as high as PP. The total decrease in u_a_ between the lowest and the highest content of post-printing waste was 6.4 kJ/m^2^, i.e., approx. 22%. The observed decrease was caused by an increase in the amount of filler in the matrix and thus also in the cross-section of the sample (Figure 7), which translated into lower polymer content and lowered the impact strength of the entire system. The large scatter of the obtained results suggests, however, that the distribution of the filler particles was heterogeneous.

The post-printing waste did not change the thermomechanical characteristics of the polymer. The PP modulus of elasticity at 30 °C (E’_30_) determined during the three-point bending DMA test was 1225 MPa and decreased with increasing temperature, reaching 132 MPa at 150 °C, i.e., just before the material melting process. The thermomechanical curves with the recorded E’ values for materials containing post-printing waste were similar to the values of pure PP, regardless of the amount of filler in the polymer matrix. Thus, the obtained materials retained the PP elasticity even with the highest content of post-printing waste. All results of E’ measurements at different temperatures are presented in Table 2.

**Figure 8:**
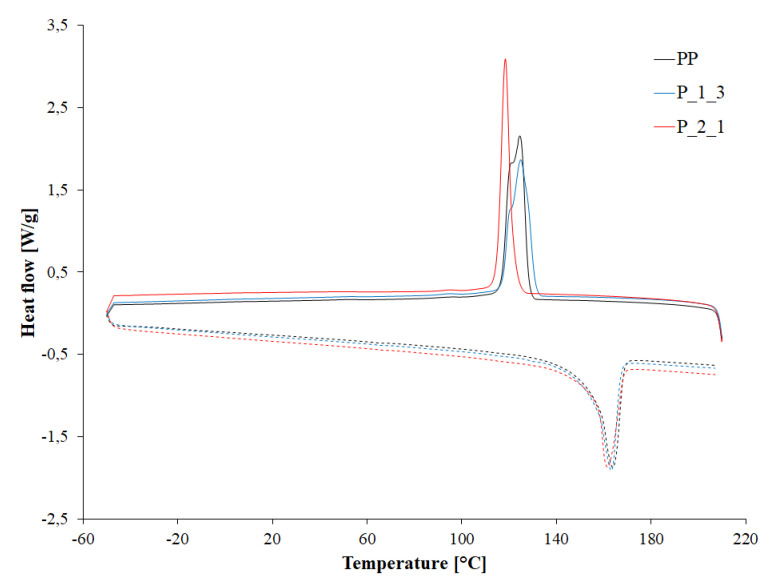
DSC curves of cooling (solid line) and heating (dashed line) of selected samples.

Post-printing waste influenced the course of phase transformations in the tested materials, i.e., changed their temperatures and intensity (Figure 8). With the lowest waste content, the thermal characteristics of the P_1_3 sample are still close to the thermal characteristics of pure PP. Only a slight decrease in the intensity of the melting process by 8 J/g is visible, which suggests a slightly lower content of the crystalline phase, which results in a reduction in the calculated degree of crystallinity by 4.7 units. On the other hand, the introduction of a larger amount of filler caused a clear decrease in the crystallization temperature (T_c_) by a maximum of approx. 6 °C for the P_2_1 sample, i.e., by approx. 5% compared to the value obtained for the PP sample.

The nature of the crystallization process also changed. At lower waste contents, the recorded peak of the crystallization process was much lower but wider than the peak recorded at the highest filler content. The enthalpy change of the crystallization process (ΔH_c_) was also higher. With the increase in the content of waste, the ΔH_c_ value decreased by 14.3 J/g, from 95.1 J/g for the P_1_3 sample to 80.8 J/g for the P_2_1 sample. Thus, the waste content affects the structure and amount of the crystalline phase formed in PP. With a lower waste content, the formed crystallites are probably larger, and there are more of them. Changes in the crystallization process are influencing the structure and quantity of the crystalline phase and obviously translate into the recorded melting process and the calculated degree of crystallinity. As the filler content increases, the melting point (T_m_) of the materials slightly decreases. The Tm decrease is approx. 2 °C when comparing the PP sample and the P_2_1 sample. The decrease in the melting enthalpy (ΔH_m_), and thus the degree of calculated crystallinity (X_c_), was much more pronounced. The ΔH_m_ value decreased by 15.6 J/g comparing the PP and P_2_1 sample. Thus, the degree of crystallinity of the tested materials decreased from 47.7 to 36.2%. All results of DSC are presented in Table 3.

**Figure 9:**
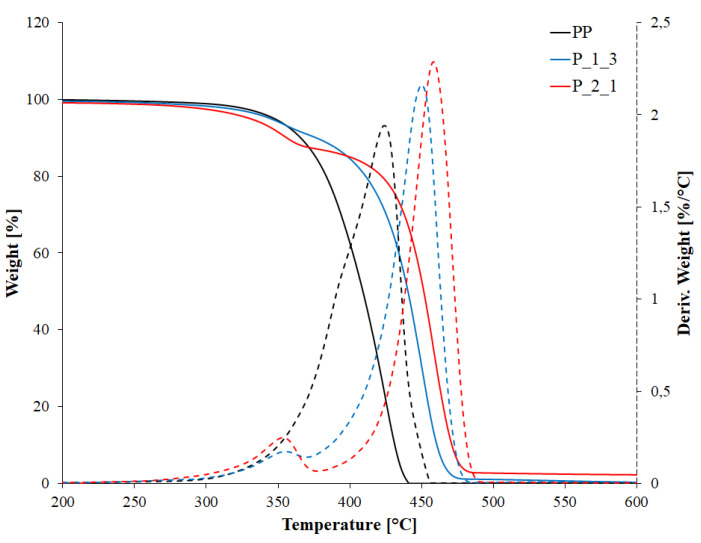
TG (solid line) and DTG (dashed line) curves of selected samples.

After introducing the post-printing waste into the polymer matrix, changes in the thermal resistance of the obtained materials were observed. Figure 9 shows the thermogravimetric curves of selected samples, while Table 4 summarizes the results of the determined thermal parameters.

As shown in Figure 9, the introduction of post-printing waste into the polymer matrix changes the nature of the degradation process from a one-stage (PP sample) to a two-stage (samples with waste). After introducing the filler, an additional degradation step appears in the range from 320 to 380 °C with the maximum decomposition rate (T_max1_) at approx. 355 °C, which becomes more significant the higher the content of waste in the polymer matrix. 

The occurrence of an additional degradation stage indirectly contributes to the reduction in the thermal resistance of materials containing waste, which is determined based on the 5% loss temperature of the sample mass (T_5%_). The thermal resistance of materials dropped from 347.5 °C for a PP sample to 344.8 °C for the P_1_3 sample and 330.6 °C for the P_2_1 sample. Therefore, the decrease in thermal resistance depended on the amount of filler in the polymer matrix. With the lowest post-printing waste content, the decrease in thermal resistance was insignificant: less than 3 °C. Increasing the waste content causes a significant reduction in thermal resistance, as the recorded value of T_5%_ decreased by 17 °C in relation to the value of pure polymer. The reduction in the recorded T_5%_ value is caused by the overlapping of the maximum waste degradation rate at the beginning of the matrix degradation, so the higher the content of the filler, the sooner the loss of 5% of the sample weight was achieved and the contractual thermal resistance of the material changed. Regardless of the thermal resistance of the tested materials, their degradation temperature significantly exceeded the typical temperatures of the use of polymer materials, and therefore, developed materials can be successfully used in most typical applications.

## 4. Conclusions

Based on the conducted research, the following can be concluded:

- The total decrease in the melt flow rate after adding the greatest amount of filler, i.e., twice the volume excess of filler, was 2.2 g/10 min, which is 20% of the value of pure polypropylene.
- The greatest decrease in the tensile strength of polypropylene after adding the post-printing waste was 4.5 MPa, i.e., approx. 19% of the value of pure polymer. This result was obtained with the highest degree of filling of the composite.
- The obtained deformation drop was the same regardless of the volumetric content of the filler in the matrix. The applied post-printing waste reduces the elongation at maximum stress and elongation at break values to the level of approx. 5%, while the values for pure polypropylene were 8.1 and 35.9%, respectively.
- The total decrease in unnotched impact strength between the lowest and the highest content of post-printing waste was 6.4 kJ/m^2^, i.e., approx. 22%.
- The introduction of post-printing waste into the polypropylene matrix did not change the thermomechanical characteristics of the polymer.
- The degree of crystallinity of the tested materials decreased from 47.7% for pure polypropylene to 36.2% for the material containing a double excess of printing waste.
- The introduction of the post-printing waste into the matrix causes a reduction in thermal resistance, as the registered value of the 5% weight loss temperature decreased by 17 °C in relation to the value of pure polypropylene.

Thus, the introduction of post-printing waste causes the deterioration of the characteristics of the obtained materials, but the decrease is fully acceptable in terms of the planned applications of the new composites. It should be remembered that the main purpose of the research described in the article was to eliminate post-production waste generated during the production of labels, which was achieved by using them as a filler for the polymer. Better properties of composites containing printing waste could probably be obtained after prior modification of the waste, so this will be the subject of further research.

## Figures and Tables

**Table 1:** Samples designations.

Sample	Filler: Matrix Volume Ratio	Mass Concentration of Filler [wt.%]
PP	-	-
P_1_3	1:3	1.9
P_1_2	1:2	2.5
P_1_1	1:1	5.1
P_2_1	2:1	10.3

**Table 2:** Results of dynamic mechanical analysis (DMA) tests of individual samples.

Sample	E’_30_ (MPa)	E’_60_ (MPa)	E’_90_ (MPa)	E’_150_ (MPa)
PP	1225	759	416	132
P_1_3	1207	782	432	142
P_1_2	1247	779	431	142
P_1_1	1326	791	443	143
P_2_1	1194	744	426	148

**Table 3:** The results of the differential scanning calorimetry (DSC) for individual samples.

Sample	Cooling		Heating		
	T_c_ [°C]	ΔH_c_ (J/g)	T_m_ (°C)	ΔH_m_ (J/g)	X_c_ (%)
PP	124.5	97.4	163.7	98.8	47.7
P_1_3	124.8	95.1	163.1	90.8	43.0
P_1_2	119.0	80.1	161.5	85.6	40.3
P_1_1	119.0	81.8	161.5	83.1	38.1
P_2_1	118.4	80.8	161.4	83.2	36.2

**Table 4:** The results of the thermogravimetric analysis (TG) of individual samples.

Sample	T_5%_ (°C)	T_50%_ (°C)	T_95%_ (°C)	T_max1_ (°C)	T_max2_ (°C)
PP	347.5	409.1	434.8	-	423.9
P_1_3	344.8	440.5	465.4	353.6	450.1
P_1_2	343.9	450.1	473.6	354.0	455.4
P_1_1	340.0	440.7	467.9	356.5	450.8
P_2_1	330.6	451.5	476.6	353.2	458.3

## Data Availability

Not applicable.

## References

[1] Das O., Babu K., Shanmugam V., Sykam K., Tebyetekerwa M., Neisiany R.E., Försth M., Sas G., Gonzalez-Libreros J., Capezza A.J. (2022). Natural and Industrial Wastes for Sustainable and Renewable Polymer Composites. Renew. Sustain. Energy Rev..

[2] Bilitewski B. (2012). The Circular Economy and Its Risks. Waste Manag..

[3] Zhou Y., Stanchev P., Katsou E., Awad S., Fan M. (2019). A Circular Economy Use of Recovered Sludge Cellulose in Wood Plastic Composite Production: Recycling and Eco-Efficiency Assessment. Waste Manag..

[4] Demirbas A. (2011). Waste Management, Waste Resource Facilities and Waste Conversion Processes. Energy Convers. Manag..

[5] Amasuomo E., Baird J. (2016). The Concept of Waste and Waste Management. J. Manag. Sustain..

[6] Basalp D., Tihminlioglu F., Sofuoglu S.C., Inal F., Sofuoglu A. (2020). Utilization of Municipal Plastic and Wood Waste in Industrial Manufacturing of Wood Plastic Composites. Waste Biomass Valorization.

[7] Quitadamo A., Massardier V., Valente M. (2019). Eco-Friendly Approach and Potential Biodegradable Polymer Matrix for WPC Composite Materials in Outdoor Application. Int. J. Polym. Sci..

[8] Teuber L., Osburg V.S., Toporowski W., Militz H., Krause A. (2016). Wood Polymer Composites and Their Contribution to Cascading Utilisation. J. Clean. Prod..

[9] Lewandowski K., Piszczek K., Zajchowski S., Mirowski J. (2016). Rheological Properties of Wood Polymer Composites at High Shear Rates. Polym. Test..

[10] Wei L., McDonald A.G., Freitag C., Morrell J.J. (2013). Effects of Wood Fiber Esterification on Properties, Weatherability and Biodurability of Wood Plastic Composites. Polym. Degrad. Stab..

[11] Friedrich D. (2021). Thermoplastic Moulding of Wood-Polymer Composites (WPC): A Review on Physical and Mechanical Behaviour under Hot-Pressing Technique. Compos. Struct..

[12] Essabir H., Hilali E., Elgharad A., El Minor H., Imad A., Elamraoui A., Al Gaoudi O. (2013). Mechanical and Thermal Properties of Bio-Composites Based on Polypropylene Reinforced with Nut-Shells of Argan Particles. Mater. Des..

[13] Laaziz S.A., Raji M., Hilali E., Essabir H., Rodrigue D., Bouhfid R., Qaiss A.E.K. (2017). Bio-Composites Based on Polylactic Acid and Argan Nut Shell: Production and Properties. Int. J. Biol. Macromol..

[14] Leszczyńska M., Ryszkowska J., Szczepkowski L. (2020). Rigid Polyurethane Foam Composites with Nut Shells. Polimery.

[15] Okonkwo E.G., Anabaraonye C.N., Daniel-Mkpume C.C., Egoigwe S.V., Okeke P.E., Whyte F.G., Okoani A.O. (2020). Mechanical and Thermomechanical Properties of Clay-Bambara Nut Shell Polyester Bio-Composite. Int. J. Adv. Manuf. Technol..

[16] Sun X., He M., Li Z. (2020). Novel Engineered Wood and Bamboo Composites for Structural Applications: State-of-Art of Manufacturing Technology and Mechanical Performance Evaluation. Constr. Build. Mater..

[17] Lokesh P., Surya Kumari T.S.A., Gopi R., Loganathan G.B. (2020). A Study on Mechanical Properties of Bamboo Fiber Reinforced Polymer Composite. Mater. Today Proc..

[18] Adediran A.A., Akinwande A.A., Balogun O.A., Olasoju O.S., Adesina O.S. (2021). Experimental Evaluation of Bamboo Fiber/Particulate Coconut Shell Hybrid PVC Composite. Sci. Rep..

[19] Mousavi S.R., Zamani M.H., Estaji S., Tayouri M.I., Arjmand M., Jafari S.H., Nouranian S., Khonakdar H.A. (2022). Mechanical Properties of Bamboo Fiber-Reinforced Polymer Composites: A Review of Recent Case Studies. J. Mater. Sci..

[20] Agunsoye J.O., Isaac T.S., Samuel S.O. (2012). Study of Mechanical Behaviour of Coconut Shell Reinforced Polymer Matrix Composite. J. Miner. Mater. Charact. Eng..

[21] Agunsoye J.O., Odumosu A.K., Dada O. (2019). Novel Epoxy-Carbonized Coconut Shell Nanoparticles Composites for Car Bumper Application. Int. J. Adv. Manuf. Technol..

[22] Nadzri S.N.I.H.A., Sultan M.T.H., Shah A.U.M., Safri S.N.A., Talib A.R.A., Jawaid M., Basri A.A. (2020). A Comprehensive Review of Coconut Shell Powder Composites: Preparation, Processing, and Characterization. J. Thermoplast. Compos. Mater..

[23] Obasi H.C., Mark U.C., Mark U. (2021). Improving the Mechanical Properties of Polypropylene Composites with Coconut Shell Particles. Compos. Adv. Mater..

[24] Sundarababu J., Anandan S.S., Griskevicius P. (2021). Evaluation of Mechanical Properties of Biodegradable Coconut Shell/Rice Husk Powder Polymer Composites for Light Weight Applications. Mater. Today Proc..

[25] Bledzki A.K., Mamun A.A., Volk J. (2010). Physical, Chemical and Surface Properties of Wheat Husk, Rye Husk and Soft Wood and Their Polypropylene Composites. Compos. Part A Appl. Sci. Manuf..

[26] Arjmandi R., Hassan A., Majeed K., Zakaria Z. (2015). Rice Husk Filled Polymer Composites. Int. J. Polym. Sci..

[27] Muthuraj R., Lacoste C., Lacroix P., Bergeret A. (2019). Sustainable Thermal Insulation Biocomposites from Rice Husk, Wheat Husk, Wood Fibers and Textile Waste Fibers: Elaboration and Performances Evaluation. Ind. Crops Prod..

[28] Bisht N., Gope P.C., Rani N. (2020). Rice Husk as a Fibre in Composites: A Review. J. Mech. Behav. Mater..

[29] Suhot M.A., Hassan M.Z., Aziz S.A., Md Daud M.Y. (2021). Recent Progress of Rice Husk Reinforced Polymer Composites: A Review. Polymer.

[30] Wilpiszewska K., Antosik A.K. (2022). Effect of Grain Husk Microfibers on Physicochemical Properties of Carboxymethyl Polysaccharides-Based Composite. J. Polym. Environ..

[31] Berger C., Mattos B.D., Amico S.C., de Farias J.A., Coldebella R., Gatto D.A., Missio A.L. (2020). Production of Sustainable Polymeric Composites Using Grape Pomace Biomass. Biomass Convers. Biorefinery.

[32] Morinaga H., Haibara S., Ashizawa S. (2021). Reinforcement of Bio-Based Network Polymer with Wine Pomace. Polym. Compos..

[33] Aljnaid M., Banat R. (2021). Effect of Coupling Agents on the Olive Pomace-Filled Polypropylene Composite. E Polym..

[34] Mirowski J., Oliwa R., Oleksy M., Tomaszewska J., Ryszkowska J., Budzik G. (2021). Poly(Vinyl Chloride) Composites with Raspberry Pomace Filler. Polymer.

[35] Hiremath P., Shettar M., Shankar M.C.G., Mohan N.S. (2018). Investigation on Effect of Egg Shell Powder on Mechanical Properties of GFRP Composites. Mater. Today Proc..

[36] Owuamanam S., Cree D. (2020). Progress of Bio-Calcium Carbonate Waste Eggshell and Seashell Fillers in Polymer Composites: A Review. J. Compos. Sci..

[37] Sakthi Balan G., Santhosh Kumar V., Rajaram S., Ravichandran Ramakrishnan M.K. (2020). Investigation on Water Absorption and Wear Characteristics of Waste Plastics and Seashell Powder Reinforced Polymer Composite. J. Tribol..

[38] Vasanthkumar P., Balasundaram R., Senthilkumar N., Palanikumar K., Lenin K., Deepanraj B. (2022). Thermal and Thermo-Mechanical Studies on Seashell Incorporated Nylon-6 Polymer Composites. J. Mater. Res. Technol..

[39] Hayta P., Oktav M. (2019). The Importance of Waste and Environment Management in Printing Industry. Eur. J. Eng. Nat. Sci..

[40] Carlos Alberto P.J., Sonia Karina P.J., Francisca Irene S.A., Adrielly Nahomee R.Á. (2021). Waste Reduction in Printing Process by Implementing a Video Inspection System as a Human Machine Interface. Procedia Comput. Sci..

[41] Medeiros D.L., Braghirolli F.L., Ramlow H., Ferri G.N., Kiperstok A. (2019). Environmental Improvement in the Printing Industry: The Case Study of Self-Adhesive Labels. Environ. Sci. Pollut. Res..

[42] Lanyi F.J., Wenzke N., Kaschta J., Schubert D.W. (2020). On the Determination of the Enthalpy of Fusion of α-Crystalline Isotactic Polypropylene Using Differential Scanning Calorimetry, X-Ray Diffraction, and Fourier-Transform Infrared Spectroscopy: An Old Story Revisited. Adv. Eng. Mater..

